# Role of SIRT1 in Chemoresistant Leukemia

**DOI:** 10.3390/ijms241914470

**Published:** 2023-09-23

**Authors:** Guadalupe Rosario Fajardo-Orduña, Edgar Ledesma-Martínez, Itzen Aguiñiga-Sanchez, Benny Weiss-Steider, Edelmiro Santiago-Osorio

**Affiliations:** 1 Hematopoiesis and Leukemia Laboratory, Research Unit on Cell Differentiation and Cancer, Faculty of High Studies Zaragoza, National Autonomous University of Mexico, Mexico City 09230, Mexico; guadalupefajardo@hotmail.com (G.R.F.-O.);; 2 Department of Biomedical Sciences, School of Medicine, Faculty of High Studies Zaragoza, National Autonomous University of Mexico, Mexico City 56410, Mexico

**Keywords:** sirtuins, AML, CML, aberrant epigenetic

## Abstract

Leukemias of the AML, CML, and CLL types are the most common blood cancers worldwide, making them a major global public health problem. Furthermore, less than 24% of patients treated with conventional chemotherapy (low-risk patients) and 10–15% of patients ineligible for conventional chemotherapy (high-risk patients) survive five years. The low levels of survival are mainly due to toxicity and resistance to chemotherapy or other medication, the latter leading to relapse of the disease, which is the main obstacle to the treatment of leukemia. Drug resistance may include different molecular mechanisms, among which epigenetic regulators are involved. Silent information regulator 2 homolog 1 (SIRT1) is an epigenetic factor belonging to the sirtuin (SIRT) family known to regulate aspects of chromatin biology, genome stability, and metabolism, both in homeostasis processes and in different diseases, including cancer. The regulatory functions of SIRT1 in different biological processes and molecular pathways are dependent on the type and stage of the neoplasia; thus, it may act as both an oncogenic and tumor suppressor factor and may also participate in drug resistance. In this review, we explore the role of SIRT1 in drug-resistant leukemia and its potential as a therapeutic target.

## 1. Introduction

Cellular homeostasis is based on the activation and selective repression of genes, and epigenetic modifications play a very important role that, depending on the genomic context and the specific type of modification, from exquisite homeostasis to the appearance of pathologies such as acute myeloid leukemia (AML), the most common type of blood cancer among the adult population. Silent information regulator 2 homolog 1 (Sirtuin-1 or SIRT1) is a histone deacetylase (HDAC) class III dependent on NAD+ that is linked to genome stability, apoptosis and autophagy, senescence, cell proliferation, and aging, which is why it is important both in healthy hematopoiesis and in hematologic malignancies. In this sense, it is overexpressed in primary samples of cancer patients [1,2] and has been linked to disease progression [3,4] and resistance to treatment [5] and is known to be overexpressed in drug-resistant cells of SKN-SH neuroblastoma, SaOS2 osteosarcoma, MCF7 breast cancer, A2780 ovarian, and IGROV1 cells [6]; however, different studies have shown an ambiguous role for SIRT1, also placing it as a tumor suppressor. The reason for this apparent contradictory action lies not in the biological activity of SIRT but rather in the cell type and cellular localization.

Drug resistance is a multifactorial cancer complication explained by the deregulation of epigenetic actors such as SIRT1, among other molecular mechanisms. The participation of SIRT1 in drug resistance in different types of cancer has been observed via drug metabolism, apoptosis induction, DNA damage, and repair or autophagy. In this sense, it has been suggested that SIRT1 could be key in the induction of chemoresistance in leukemia via the deregulation of p53 in AML and p53, Ku70, FoxO1, and Hsp90 in CML, nonhistone substrates deacetylated and inactivated by SIRT1 [7,8]. Although the signaling pathways that lead to chemoresistance are still being explored, there is evidence that supports the participation of SIRT1; therefore, knowing the role of SIRT1 in the induction of drug resistance in leukemia would allow us to propose treatment strategies that prevent and reverse the chemoresistant phenotype, thus contributing to improving the survival rate.

## 2. SIRT in Health

There are epigenetic mechanisms that modulate gene expression by remodeling the chromatin structure via posttranslational modifications of histone proteins. Such modifications may include but are not limited to methylation, phosphorylation, ubiquitination, and acetylation/deacetylation of histones; these last modifications are the most common. Histone acetylation is the addition of an acetyl group to lysine residues in protruding histone tails, while deacetylation is the process of removing the acetyl group. Acetylation is executed by “histone acetyltransferases” (HATs) and deacetylation by protein histone deacetylases (HDACs), among which are sirtuins (SIRTs). SIRTs are a family of seven proteins in mammals (SIRT1 to SIRT7), differing in subcellular localization and biological functions [9]. It has been found that the nucleus expresses SIRTs 1, 6, and 7, while the mitochondria express SIRTs 3, 4, and 5; the expression of SIRT2 is mainly found in the cytosol, although it can also be found in the nucleus under conditions of cellular stress [10,11]. SIRTs require the removal of acetyl groups on lysine residues and the presence of the cofactor nicotinamide adenine dinucleotide (NAD+) [12]. Nutritional and environmental factors are known to significantly affect intracellular NAD+ levels such that decreasing levels of cellular NAD+ can alter the activities of SIRTs and alter chromatin structure and mitochondrial metabolism, leading to increased oxidative stress, inflammation, and cell damage [13,14].

Alternatively, SIRTs can deacetylate nonhistone proteins, thereby participating in different aspects of cell regulation, such as the cell cycle, growth, energy metabolism, cell differentiation, and apoptosis, both in health and disease [15]; here, we will only refer to SIRT1 (Table 1).

In normal tissue homeostasis, SIRT1′s main functions are related to protection against tissue degeneration and age-related diseases related to cellular stress and oxidation. In this way, SIRT1 regulates the expression of molecules related to cell proliferation, apoptosis, and the stress response, such as c-MYC, FOXO3a, RB1, KU70, E2F1 [1,16,17], Hif, HSF1, and FOXO1–O4, to regulate cell growth, apoptosis, and the stress response [1,16,17,18,19,20]. In response to cell damage and oxidative stress, SIRT1 deacetylates AKT, MAPK, NRF2, P53, NF-κB, Ku70, XPA, and NBS1 [16,21,22,23,24] and interacts with members of the MYST acetyltransferase family, such as hMOF and TIP60 [25].

## 3. SIRT1 in Normal Hematopoiesis

Among the different isoforms, SIRT1 is the most studied, most likely due to its involvement in the homeostatic regulation of different organs and tissues, both in the adult stage and during embryonic development [26]. Although it was originally associated with longevity [11,27,28], it plays a role in regulating metabolism as well as in DNA repair in the inflammatory and stress response [29,30,31].

Through in vitro studies, it has been found that during human embryonic development, SIRT1 activates the expression of hemoglobin genes in erythroblasts, thus establishing primitive hematopoiesis [32,33]. The importance of SIRT1 in hematopoiesis is evident when SIRT1−/− mESCs aberrantly differentiate into erythroid progenitors and produce reduced numbers of progenitors under ex vivo hypoxia [34]. In this sense, the knockdown of SIRT1 compromises hematopoiesis, increasing the HSC cycle and depletion in response to stress; in addition, it induces anemia, myeloid expansion, lymphoid depletion, accumulation of DNA damage, and reduced production of erythropoietin [35,36].

SIRT1 deficiency in mouse embryonic stem cells compromises their differentiation capacity [10], raising the concern that adult hematopoiesis may also be compromised. In this sense, C57BL/6 Sirt1+/− mice exposed to radiation have reduced survival rates and counts of hematopoietic cells. It has been found that Sirt1−/− HSCs from C57BL6 mice escape from quiescence and exhibit greater accumulation of ROS. SIRT1′s contribution to the HSC phenotype may occur via deacetylation of the transcription factor Forkhead Box (FOXO3), which maintains quiescence and promotes self-renewal in HSCs [9,36,37].

SIRT1 plays a key role in granulocyte and macrophage homeostasis. NAMPT-treated CD34+ cells differentiate into granulocytes through autocrine expression of G-CSF and its receptor, probably via SIRT1-CCAAT/enhancer binding protein (C/EBP) [38]. Analogously, but in myeloid and neutrophil progenitors of subjects with congenital neutropenia, G-CSF stimulates the expression of NAMPT, leading to the synthesis of NAD+ and SIRT1 [39]. Likewise, SIRT1 regulates the cell cycle of macrophages and longevity pathways; in addition, it favors the polarization of macrophages toward the M2 phenotype by reducing TNF-α and IL-1β [40,41]. On the other hand, SIRT1 influences the stroma that supports hematopoiesis, since in mesenchymal stem cells (MSCs), it promotes the expression of CXCL12 by inhibiting p53 activation, thus favoring the recovery of the hematopoietic niche [42].

However, unlike C57BL/6 mice, deletion of SIRT1 in HSCs from C57BL/6NCr mice does not significantly affect stem and progenitor populations and does not affect GMP progenitor count (Lin^−^ Sca1^−^ c-Kit^+^ CD34^+^ FcγRII/III^hi^) or megakaryocytic–erythrocytic progenitors (Lin^−^ Sca1^−^ c-Kit^+^ CD34^−^ FcγRII/III) [36]. Leko et al. showed that SIRT1 does not play an essential role in the maintenance of the HSC compartment in adult C57BL/6 mice, and deletion did not affect HSC maintenance and long-term reconstitution in adult mice in the steady state [34]. Additionally, SIRT1 inhibition has only a minor impact on normal human CD34+ hematopoietic cells [42]; thus, the role of SIRT1 in adult HSCs is still controversial. The basis of this difference in the apparent importance of SIRT1 in adult mouse hematopoiesis could be found in the C57BL/6 mouse model. The Sirt1+/− phenotype involves deletion of exon 4 [34,36,43]; thus, there is homogeneity between different studies but it is not infrequently observed in the C57BL/6 mouse model. It is omitted to specify whether a particular substrain has been used. Siegfried Bezdek et al. found in C57BL/6J vs. C57BL/6 N mice significant differences in the course of skin inflammation, such as erythema, infiltration, and desquamation, between these two closely related mouse substrains. Genetic differences between C57BL/6 substrains have been described before, and the authors suggest that great caution is warranted when using C57BL/6 mice for dermatitis model studies if the genetic background between substrains is not exactly the same, as there is a high risk of false results and consequently false conclusions [44]. Whether minimal genetic differences that explain the distinct manifestations of dermatitis in different C57BL6 substrains extend to differences in SIRT1 involvement in adult mouse hematopoiesis needs to be explored.

On the other hand, several studies indicate a pathogenic role for SIRT1 in solid tumors and leukemias [45] and with the chemoresistant phenotype, but other studies suggest tumor-suppressive functions [46,47], implying that the role of SIRT1 in cancer may be context dependent, varying by the tumor type, specific oncogenes present, and mutation status of p53 or other target proteins [45].

## 4. SIRT1 in Chemoresistant Leukemia

Chemotherapy remains the main treatment for hematological malignancies despite the significant development of other cancer therapies during past decades. Depending on the mechanism of action, commonly used chemotherapeutic agents can be divided into several classes, such as antimetabolites, alkylating agents, mitotic spindle inhibitors, topoisomerase inhibitors, and others [48]. An initial favorable response to treatment can later change (acquired resistance), leading to relapse and recurrence. In addition, in some types of cancer, there is a lack of initial response to treatment, reflecting a form of intrinsic resistance (primary resistance). In any case, resistance to therapy is a major problem for fully effective therapy [49] that could be a resistance only to the inducing drug without showing cross-resistance to other drugs or resistance to multiple drugs (MDR) with different structures and mechanisms of action than the original antitumor drug [50].

The emergence of resistance is a multifactorial phenomenon explained by a variety of molecular mechanisms that include various genes or proteins with altered expression or function, leading to the reduced efficacy of anticancer agents. In this sense, the main causes of chemoresistance in hematological malignancies include the presence of efflux pumps, changes in apoptotic and metabolic signaling pathways, microenvironmental alterations, miRNAs, DNA repair systems, cell cycle arrest, and quiescence in leukemic stem cells [51]. Genomic instability evidenced by the appearance of point mutations, chromosomal rearrangements, etc. explains the appearance of the chemoresistant phenotype; however, there has also been evidence that epigenetic regulators are involved in this phenomenon.

SIRT1, in addition to participating in healthy hematopoiesis and in the maintenance of hematological malignancies such as acute myeloid leukemia (AML), chronic myelogenous leukemia (CML), mixed phenotype acute leukemia (MPAL), T-cell acute lymphoblastic leukemia (T-ALL), chronic lymphocytic leukemia (CLL), and diffuse large B-cell lymphoma (DLBCL), is increased [3,4,52]. In LSC populations in AML and CML, SIRT1 has been directly linked with drug resistance in addition to maintaining survival and growth [5] (Table 2).

### 4.1. SIRT1 in Chemoresistant CML

SIRT1 seems to be a key regulator of drug resistance in malignancies such as CML [61]. Resistance to imatinib results in most cases from amplification of the BCR-ABL gene or from point mutations in the same gene, leading to overexpression of the BCR-ABL protein [62,63]. BCR-ABL transcriptionally activates SIRT1 as a stress response gene in hematopoietic progenitor cells, promoting the deacetylation of multiple substrates, such as p53, Ku70, FoxO1, and Hsp90, resulting in reduced apoptosis and the induction of proliferation, oncogenic transformation, and leukemogenesis [64].

In imatinib-resistant CML, it has been shown that the knockdown of BCR-ABL decreased the levels of SIRT1 and FoxO1 proteins and increased the levels of Ac-FoxO1 and p53 proteins, indicating that SIRT1 is downstream of BCR-ABL [55]. The model of KCL-22 resistance to Bcr-Abl inhibition shows that SIRT1 is a key intermediary in promoting the acquisition of gene mutations leading to drug resistance [53]. KCL-22 cells originated from a patient with CML in blast crisis who was sensitive to imatinib treatment. In the presence of imatinib, such patients acquire resistance via the T315I BCR-ABL mutation, but when treated with SIRT1 inhibitors (sirtinol, nicotinamide, and tenovin-6), relapse is blocked. Whereas the combination of sirtinol and imatinib increased cell death, tenovin-6 blocked relapse at concentrations as low as 1 μM, which was below the concentration required to increase imatinib-mediated cell killing. Similarly, although nicotinamide blocked cell relapse, it was not enough to potentiate cell death. Treatment with nilotinib and dasatinib (two second-generation BCR-ABL inhibitors) induces the acquisition of the T315I BCR-ABL mutation, leading to relapse, but SIRT1 inhibition with tenovin-6 or sirtinol blocks recurrence [53].

In addition to BCR-ABL and SIRT1, high levels of expression of Lyn, a member of the Src family kinases (SFKs) not affected by imatinib, have been detected. In some patients, disease progression is mediated by this kinase in a BCR-ABL-independent mechanism [55,65].

The acquisition of resistance via Lyn correlates with decreased expression of BCR-ABL, as has been shown in Bcr-Abl-positive CML cells cultured in the continuous presence of imatinib [32]. In this regard, Bcr-Abl-negative imatinib-resistant K562 cells showed marked declines in mRNA and protein levels and activity of Bcr-Abl that are associated with low AKT kinase and STAT5 activity [66]. In K562 cell variants, imatinib resistance was associated with low levels of BCR-ABL and its tyrosine kinase activity (a phenomenon also detected in chemoresistant CML patients). Unlike parental K562 cells, imatinib-resistant variants showed a reduction in levels of Bcr-Abl, without mutations in the Abl kinase domain but upregulation of SIRT1 and class I HDACs (HDAC1, 2, and 3) as well as downregulation of histone acetyltransferase CBP/p300 and PCAF. Interestingly, the nonhistone proteins p53 and Hsp90 exhibited aberrant acetylation and a concurrent upregulation of antiapoptotic molecules such as Bcl-2 and cytoplasmic Ku70 and downregulation of Bax and p21; thus, the authors suggest that deregulated acetylation of nonhistones via SIRT1 is an alternative mechanism for the Bcr-Abl-independent form of imatinib resistance [8].

Hsp90 is the major mammalian chaperone protein that interacts with oncogenic proteins and cochaperones in cancer cells and is thus required by BCR-ABL for stabilization and maturation. Hsp90 inhibitors destabilize the binding of BCR-ABL protein, leading to its degradation via the ubiquitin-proteasome pathway [67]. Inhibition of HSP90 overcomes resistance to chemotherapy and radiotherapy in some cancers [68]; therefore, it is an important pharmaceutical target in cancer therapy [69]. The expression of multidrug transporters is a key feature of the acquisition of chemoresistance; thus, deletion of transporters such as BCRP and P-gp has been shown to increase sensitivity to anticancer drugs [70]. In imatinib-resistant K562 cells expressing high levels of CD44 and CSC-related molecules—such as Oct4, CD34, β-catenin, c-Myc, mutant p53, BCRP, and P-glycoprotein (P-gp)—inhibition of SIRT1 blocked BCRP and P-glycoprotein-mediated efflux activity; further, SIRT1 depletion by siRNA or SIRT1 inhibitors, such as amurensin G and EX527, caused significant downregulation of Hsps as well as these CSC-related molecules. This led to the sensitization of CD44-high K562 cells to 17-AAG and AUY922, both Hsp90 inhibitors; thus, SIRT1 inhibition could reverse the resistance of CD44-high K562 cells to Hsp90 inhibitors [54].

Cancer drug combinations are an attractive approach to overcome resistance and deliver new treatments. Combination therapy with tyrosine kinase inhibitors (TKIs), the main therapeutic drug regimens for CML, and histone deacetylase inhibitors has been tested in CML to reduce drug resistance by eliminating quiescent leukemic stem cells [71,72]. Nicotinamide (NAM), a noncompetitive inhibitor of SIRT1, plus doxorubicin (DOX) inhibited the proliferation and apoptotic resistance of DOX-resistant K562 cells, promoting the activation of caspase-3 and PARP. Additionally, in in vivo experiments, DOX in combination with NAM inhibited the growth of K562R xenografts more efficiently than either NAM or DOX alone [56]. This combination strategy of drugs directed at epigenetic remodelers is in a very early phase, so the data are limited. More studies are needed, especially to explore possible drug interactions. However, it represents an area of opportunity for treatment against resistance that should not be dismissed.

### 4.2. SIRT1 in Chemoresistant AML

SIRT1 is selectively overexpressed in primary human FLT3-ITD AML of patients with induction failure, relapse, or persistent disease status. SIRT1 overexpression is related to enhanced expression of the USP22 deubiquitinase induced by c-MYC, leading to reduced SIRT1 ubiquitination and enhanced stability, but inhibition of SIRT1 expression or activity reduced the growth of FLT3-ITD AML LSCs and significantly enhanced TKI-mediated killing of the cells. SIRT1 expression correlated with increased resistance to targeted therapy of FLT3-mutated AML cells [60], and SIRT1 pharmacological targeting inhibited cell growth and sensitized AML cells to treatment with tyrosine kinase inhibitors and chemotherapy via restoration of p53 activity [1].

FOXP1, a subtype of the Forkhead Box (FOX) transcription factor, is an essential regulator of HSPC maintenance and AML cell growth and survival that does so by repressing p21CIP and p27KIP cell cycle inhibitors [73]. Moreover, FOXP1 has been proposed as an adverse prognostic factor in intensively treated AML patients who received autologous stem cell transplant at remission; AML patients with elevated FOXP1 gene expression at diagnosis had a significantly shorter progression-free and overall survival after intensive induction chemotherapy and autoSCT [74]. FOXP1 enhances the expression of SIRT1, a well-known negative regulator of p53 activity, thus promoting cell survival under stress conditions in many cells, including AML and CML stem cells [75]. AML cells overexpressing SIRT1 via an inducible lentiviral vector were treated with increasing concentrations of daunorubicin, and FOXP1 activity protected AML cells against cytotoxic treatment; thus, the level of FOXP1 expression was correlated with increased resistance to daunorubicin in AML cells that overexpress SIRT1 [57].

Interestingly, SIRT1 works as a cellular metabolism sensor that selectively activates PGC-1α (a transcription factor coactivator that influences most cellular metabolic pathways) and autophagy actors [5]. Thus, SIRT1 plays a critical role in stress-induced hematopoiesis, metabolic reprogramming, and maintenance of AML/CML leukemic stem progenitor cell (LSC) regenerative potential; it contributes to the maintenance of CML LSCs following targeted therapy [57].

When the mRNA and protein expression levels of Sirt1 and PGC-1a were compared in the cytarabine-resistant Kasumi-1 cell line and its parental cells, higher Sirt1 and PGC-1a expression was found in the cytarabine-resistant cell lines; thus, the authors suggest that high coexpression of Sirt1 and PGC-1a may mediate drug resistance in these AML cells [5].

Among the different biological effects induced by SIRT1 is increased autophagy [17] by the deacetylation of lysine residues on crucial autophagy proteins such as Beclin1, ATG5, ATG7, and LC3 [76].

Autophagy is a dynamic process of protein degradation that is typically observed during nutrient deprivation and genotoxic stress. This process protects cells from harmful metabolic conditions by removing damaged organelles and recycling breakdown products in normal cells and thus may play a protective role against cancer, but excessive autophagy induced by metabolic and therapeutic stress may have the opposite effect and promote tumor cell survival with defects in apoptosis [77,78,79,80,81].

There is evidence that SIRT1 is required for the induction of autophagy in hepatocytes [82] to protect cardiomyocytes from hypoxic stress via AMPK activation and reduced hypoxia-induced apoptosis [17]. SIRT1 enhances autophagic activity in ATDC5 chondrocyte cells [83], and in AML, SIRT1 is related to the ability to undergo autophagy under stress [58].

Apoptosis blockade is a frequent mechanism in the induction of chemoresistance, so cancer cells could maintain autophagy induction as a survival mechanism against apoptosis arrest and prolonged therapeutic stress. In coculture of AML cells with stromal cells, increased autophagy is correlated with chemoresistance to cytarabine and idarubicin, and this is reversed following Atg7 knockdown, a key molecule in autophagy vesicle elongation and involved in ubiquitin-like reactions [84].

However, the relationship between SIRT1, autophagy, and resistance to cytarabine seems to be linked to SDF-1a-CXCR4 signaling, which interacts with ATG5 and LC3. It has been shown that interactions of SDF-1a with CXCR4 promote the in vitro survival of AML cells and that treatments with SDF-1a antibodies, neutralizing antibodies against CXCR4, and CXCR4 inhibitors are effective against AML cells. SIRT1 knockdown in OCI-AML2 cells attenuated the ability to undergo autophagy under stress and enhanced cytarabine-induced apoptosis; thus, SIRT1 contributes to the chemoresistance of AML cells [58].

Tumor suppressor p53 becomes activated in response to a myriad of stresses, including DNA damage, oxidative stress, and ionizing radiation, leading to diverse cellular responses, including cell cycle arrest, apoptosis, senescence [85], and autophagy. Nuclear p53 can induce autophagy and promote apoptosis, while cytosolic p53 may act as a repressor of autophagy. Little is known about the relationship between chemoresistance, p53, and autophagy in AML, but with the MDR phenotype, both wild-type and mutated p53 could reverse MDR. Interestingly, it was also reported that MDR phenotype ovarian cancer cells use autophagy as a protective phenomenon. However, the different forms of p53 have different mechanisms of action; mutant p53 utilizes autophagy to kill MDR-positive ovarian cancer cells, while wild-type p53 inhibits autophagy and reverses MDR [86]. The inhibitory effect of p53 on autophagy provides different outcomes, either inhibiting apoptosis or promoting tumor cell growth under autophagy-inducing conditions. The association of p53 with MDR is dependent on the state of p53, the cell type, the surrounding environment, the underlying mechanism, etc.; so, in this sense, its role in autophagy in response to tumor drugs is also complex. Inhibition of autophagy by p53 in the ovarian cancer cell line with the MDR phenotype SKVCR confers a drug-sensitive phenotype [79]. However, p53-mutated cells maintain high autophagy rates under chemotherapy, and it is likely that functional wtp53 maintains autophagic homeostasis and adjusts the rate of autophagy to changing circumstances; thus, p53 is neither a positive nor negative regulator of autophagy [87].

At least 11 ABC transporters, including P-glycoprotein (P-GP/ABCB1), multidrug resistance-associated proteins (MRPs/ABCCs), and breast cancer resistant protein (BCRP/ABCG2), are involved in multidrug resistance (MDR) development [88]. Extensive studies indicate that both p53 and multidrug transporter proteins play an important role in chemoresistance; wild-type p53 downregulates the expression of multidrug-resistant proteins, while mutant p53 proteins upregulate Pgp and MRP expression [89].

The most important prognostic factor in solid tumors, such as breast cancer or osteosarcoma, is the coexpression of the p53 protein and ejection pumps, and their presence is decisive for shorter survival in locally advanced cancer [90] and poor prognosis [40]. In human vincristine (VCR)-resistant cells of leukemia/lymphoma cell lines, it has been shown that p53 may directly or indirectly mediate the expression of mdr-1 via WT1 [91] and p53 overexpression was more frequently observed in AML patients with a poor prognosis, such as those with secondary and relapsed conditions, suggesting that MDR could be associated with p53 mutations in the advanced stage of the disease, but no significant association between p53 expression and MDR has been observed in AML, ALL, and CLL, and only in CML was a significant association between p53 overexpression and MDR observed, more frequently in accelerated and myeloid blastic phases of disease [92].

In contrast, in leukemia/lymphoma cells, p53 expression was found to disappear as soon as the cells expressed the mdr-1 gene. It is known that the tumor suppressor activity of p53 is inactivated by point mutations or other types of genetic changes, such as epigenetic factors, which leads to the development of many types of malignancies; these observations lead to speculation that upon the development of drug resistance, the expression of p53 may disappear [92].

## 5. Chemoresistance in Leukemia via SIRT1, Possible Mechanisms, and Therapeutic Opportunities

**Figure 1:**
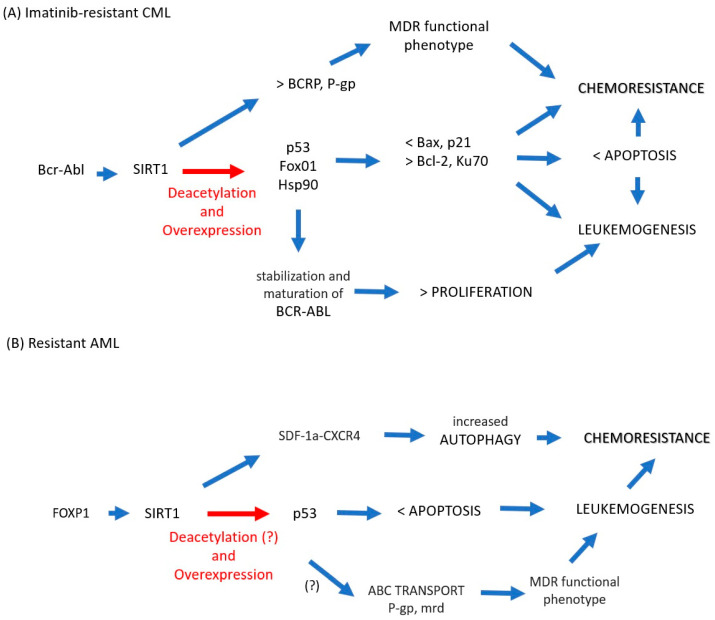
(**A**) In imatinib-resistant CML cells, BCR-ABL increases the expression of SIRT1, which induces aberrant acetylation in nonhistone substrates p53 and FoxO1, which translates into a lower expression of proapoptotic factors (Bax, p21) but at the same time upregulation of antiapoptotic molecules (Bcl-2, Ku70), leading to survival in the presence of imatinib. Hsp90 acetylation favors the stability of BCR-ABL and with it cell proliferation and the maintenance of leukemogenesis. Additionally, SIRT1 is directly linked to drug exclusion via overexpression of BCRP and P-gp. Thus, sustained proliferation, blockade of apoptosis, and drug exclusion lead to a chemoresistant phenotype. (**B**) In AML cells, FOXP1 regulates the expression of SIRT1, which correlates with greater resistance to drugs such as daunorubicin; in resistant cytarabine cells, SIRT1 is related to the induction of autophagy as a survival mechanism in cells with apoptosis arrest. In this case, although the expression of p53 is conditional regarding the type of AML and the tumor context and aberrant acetylation of p53 in AML by SIRT1 has not been shown, it is known that p53 is responsible for blocking apoptosis; thus, the metabolic stress resulting from the arrest of apoptosis could reinforce the autophagic phenotype. Finally, p53 correlates with the chemoresistant phenotype via ABC transporters, and the association of SIRT1 in this context needs to be clarified, which would allow us to propose a SIRT1–p53–chemoresistance axis. Blue arrow: increased, Red arrow: potentially increased.

The data collected for chemoresistant CML suggest that SIRT1 is involved in the acquisition of drug resistance as follows (Figure 1A). Bcr-Abl induces overexpression of SIRT1, which induces overexpression and deacetylation of various nonhistone substrates, including suppressors of tumors such as p53 and FoxO1. The reduced activity of these substrates results in the negative regulation of proapoptotic factors such as Bax and p21, but at the same time, there is a positive regulation of antiapoptotic molecules such as Bcl-2 and Ku70. This imbalance leads to survival in the presence of imatinib. However, the acetylation of Hsp90 because of SIRT1 favors the stability of BCR-ABL, cell proliferation, and the maintenance of leukemogenesis, which in turn favors genomic instability and the acquisition of secondary mutations. SIRT1 is directly linked to the exclusion of drugs via the overexpression of BCRP and P-gp, the main exclusion pumps of therapeutic xenotoxics. Thus, sustained proliferation, blockade of apoptosis, and drug exclusion lead to a chemoresistant phenotype. In AML (Figure 1B), the data are less conclusive, and much remains to be clarified, but there is evidence that FOXP1 regulates the expression of SIRT1, which correlates with a greater resistance of AML cells to drugs such as daunorubicin. The mechanism is not clear, but there is a possibility that autophagy and p53 regulation are involved as follows: SIRT1 is related to the induction of autophagy (via SDF-1/CXCR4) in cytarabine resistance. SIRT1 induces deacetylation in p53, which leads to maintaining the survival of the leukemic cell by blocking apoptosis although the molecular mechanism has not been explored. Although the expression of p53 is conditional on the type of AML and the tumor context, it is known to be responsible for blocking apoptosis; thus, the metabolic stress resulting from the arrest of apoptosis could reinforce the autophagic phenotype. Finally, although the relationship between SIRT1, p53, and MDR has not been conclusively demonstrated, lower p53 expression correlates with the chemoresistant phenotype via ABC transporters, and SIRT1 reduces p53 activity via deacetylation; therefore, there is the possibility that a SIRT1–p53–chemoresistance axis needs to be explored in the future.

**Figure 2:**
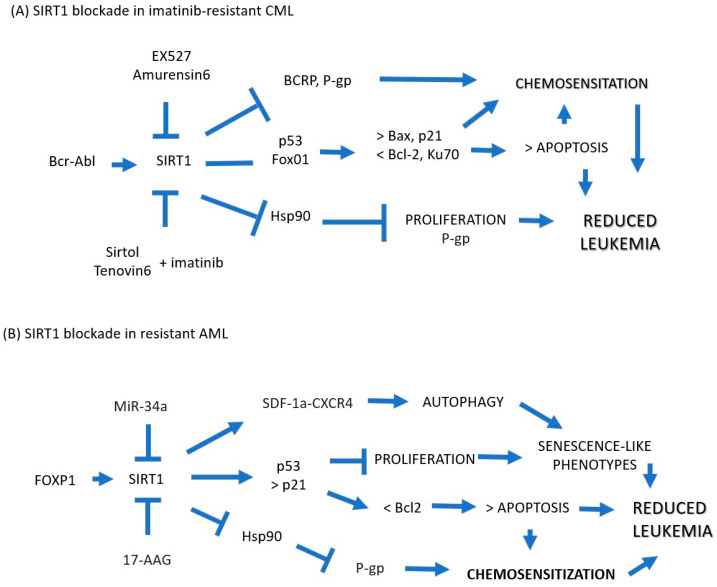
(**A**) In imatinib-resistant CML cells, blockade of SIRT1 with EX527/amurensin6 or Sirtol/Tenovin9 has been shown to be associated with blockade of recurrence and relapse, potentiation of proliferation inhibition, and induction of apoptosis in the presence of imatinib and blockade chemosensitization from the exclusion of drugs via BCRP and P-gp and reduction in the expression of Hsps and LSC-related molecules. (**B**) In AML cells, it could be explored whether SIRT1 inhibitors such as miR-34a and 17-AAG can chemosensitize cells resistant to treatment via overexpression of p53 and p51 with subsequent potentiation of apoptosis while blocking the induction of Hsp90, which protects cancer cells from apoptosis and induces drug resistance via the expression of P-gp. Blue Arrow: Increased, Blue Arrow Flat Tip: Locked.

In CML, the involvement of SIRT1 in the maintenance of leukemogenesis and in the acquisition of chemoresistance poses a window of opportunity as a therapeutic target that is already beginning to be explored (Figure 2). In imatinib-resistant CML cells, the use of SIRT1-specific blockers EX527/amurensin6 and Sirtol/Tenovin9 is associated with blockade recurrence and relapse, potentiation of proliferation inhibition, and induction of apoptosis in the presence of imatinib and chemosensitization by blockade of SIRT1. The exclusion of drugs via BCRP and P-gp reduces the expression of Hsps and LSC-related molecules. However, leukemogenesis and the acquisition of chemoresistance are the result of the alteration of multiple signaling pathways, so it is unlikely that focusing on a single molecular target is the answer against leukemia, as has already been demonstrated with the use of imatinib and its derivatives. In contrast, and as has been suggested by others, the combination of different emerging therapeutic molecules that simultaneously inhibit key molecular mechanisms of chemoresistance render leukemia cells unable to overcome chemotherapy.

Similarly, the participation of SIRT1 in the maintenance of leukemogenesis and in the acquisition of chemoresistance could be used as a therapeutic target, although little has been explored in this regard in leukemia. In this sense, it has been shown in human esophageal squamous cancer cells with a wild-type p53 gene background that miR-34a, a noncoding RNA universally expressed in normal old cells and a pivotal member of the p53 network, can inhibit cell growth accompanying the downregulation of SIRT1 and upregulation of p53/p21 and senescence-like phenotype in ECa-109 [93]. In addition, it has been shown that the activation of miR-34a can induce apoptosis and/or senescence in breast cancer cells via the downregulation of Bcl-2 and SIRT1 [60]. Therefore, it would be interesting to explore whether this noncoding RNA can chemosensitize resistant AML cells. In addition, inhibition of SIRT1 with 17-allylamino-17-demethoxy-geldanamycin (17-AAG) induces Hsp90/Hsp70 hyperacetylation and decreases the formation of the Hsp90 multichaperone complex and subsequent downregulation of P-gp in P-gp-positive human lymphoblastic leukemia MDR cell lines (CEM/VLB55–8 and CEM/VLB100 MDR) [54].

Alternatively, in a mouse model of acute promyelocytic leukemia, treatment with a CXCR4 antagonist made leukemia cells more sensitive to cytarabine and prolonged the survival of tumor-bearing mice compared to both untreated mice and mice treated with cytarabine alone [58], thus suggesting a role for CXCL12/CXCR4 inhibition in hematological malignancy treatment; therefore, there is the possibility that CXCR4 blockade could contribute to AML chemosensitization.

## 6. Conclusions

Leukemia therapy has not changed in the last 50 years, as evidenced by the sustained and widespread use of genotoxic drugs such as cytarabine and anthracyclines. With notable exceptions, drugs directed against molecular targets have provided a window of hope; however, it has been shown that patients can develop treatment-associated chemoresistance in which aberrant epigenetics play an important role. The epigenetic factor SIRT1, known to regulate different aspects of homeostasis and cancer, plays an important role in normal and neoplastic hematopoiesis. In CML and AML, there is evidence that SIRT1 is involved in the acquisition of drug resistance; in this way, it could be used as a therapeutic target. However, as we show here with SIRT1 and leukemia, there are still many pieces to be discovered. Only in this way can effective therapeutic strategies be developed capable of limiting the appearance of chemoresistance and, where appropriate, reversing chemoresistance in leukemia patients.

## Figures and Tables

**Table 1:** Nonhistone proteins deacetylated by SIRT1.

Nonhistone Proteins	Physiologic Role	Effects of Deacetylation by SIRT1
AKT	Cell damage and oxidative stress	Promotes cell proliferation, migration, and invasion but inhibits apoptosis
c-MYC	Cell proliferation, apoptosis, differentiation, and metabolism	Facilitates transactivation of c-Myv at the hTERT promoter
E2F1	Proliferation	Promoting cell proliferation
FOXO1	Cell cycle and apoptosis	Inhibition of apoptosis via FoxO1/β-catenin, promotion of FOXO target genes involved in stress resistance
FOXO3a	Proliferation, response to cellular damage and oxidative stress	Suppresses FoxO3a-mediated oxidative stress resistance, modulates the balance between antiapoptotic and apoptotic genes
KU70	Proliferation, cell damage response and oxidative stress, DNA repair, regulation of transcription and recombination	Increases repair activity, prevents apoptosis
MAPK	Response to cell damage and oxidative stress	Represses MAPK signaling pathways
NF-κB	Regulation of cell proliferation, apoptosis, inflammation, immune response, oxidative stress	Suppresses the transcription activity of NF-κB, reducing the production of oxygen radicals and thereby resisting oxidative stress injury, blocking oxidative stress damage
NRF2	Cell damage and oxidative stress	Regulates antioxidant gene expression
P300	Rate-limiting transcriptional cointegrator of transcription factors either to activate or to repress transcription	Important integration point during metabolism and cellular differentiation
P53	Stress response transcription factor, cell cycle, DNA repair, and apoptosis	Prevents DNA repair and is an apoptosis-inhibiting signaling pathway (reducing expression of p53, p21, and caspase-3)
PGC-1α	Coactivator of peroxisome proliferator-activated receptor-γ, which can block oxidative stress damage by scavenging excess ROS	Activates PGC-1α through deacetylation, scavenges ROS caused by oxidative stress, and alleviates oxidative stress injury
RB	DNA synthesis and cell cycle control	Promotes cell proliferation
TIP60	Cell growth and arrest, apoptosis, and DNA repair	Inhibits autoacetylation and HAT activity

**Table 2:** SIRT1 involvement in drug resistance in leukemia.

Type Leukemia	Drug Status	Molecules Related to Chemoresistance	Reference
CML			
K562 cells	Imatinib-resistant	Hsp90, p53, and Ku70	[8]
KCL-22 cells	Imatinib-resistant	T315I BCR-ABL	[53]
K562 cells	Imatinib-resistant	Hsps, BCRP and P-gp	[54]
K562 cells	Imatinib-resistant	Lyn kinase	[55]
K562 cells	Doxorubicin-resistant	No data	[56]
AML			
MOLM-14 cells OCI-AML2 cells KASUMI-1 cells	Daunorubicin-resistant	FOXP1	[57]
OCI-AML2 cells	Citarabina-resistant	SDF1α–CXCR4	[58]
Kasumi-1 cells	Citarabina-resistant	PGC-1α	[59]
FLT3-ITD cells	Induction failure relapse or persistence	USP22 and c-MYC	[60]
